# The clinical value of PLR, NLR, and MLR in predicting mortality risk in hospitalized patients

**DOI:** 10.3389/fphar.2025.1736947

**Published:** 2025-12-03

**Authors:** Guoxiang Fang, Yunfei Mo, Yangwen Bao, Chunbiao Gao, Qiuya Pan, Tongbo Lu

**Affiliations:** 1 Department of Clinic Laboratory, Changzhou De’an Hospital, Changzhou, Jiangsu, China; 2 Department of Clinic Laboratory, Children’s Hospital of Changzhou, Changzhou, Jiangsu, China; 3 Department of Rehabilitation, Changzhou De’an Hospital, Changzhou, Jiangsu, China

**Keywords:** platelet/lymphocyte ratio, neutrophil/lymphocyte ratio, monocyte/lymphocyte ratio, hospitalized patients mortality, mortality risk

## Abstract

**Background:**

To analyze the trends and clinical significance of the platelet/lymphocyte ratio (PLR), neutrophil/lymphocyte ratio (NLR), and monocyte/lymphocyte ratio (MLR) in hospitalized patients prior to death.

**Method:**

A retrospective analysis was conducted on the PLR, NLR, and MLR of 341 hospitalized patients prior to death from January 2016 to October 2020 at Changzhou De’an Hospital, using inpatients with kidney disease and patients in rehabilitation as case-control groups, and hospital staff as a healthy control group, to statistically analyze relevant clinical data and test results.

**Results:**

Significant differences were observed in the PLR, NLR, and MLR prior to death in hospitalized patients compared to those with kidney disease, those in recovery, and the healthy control group (*P* < 0.05). No statistically significant differences were found among different genders and age groups. The PLR, NLR, and MLR showed significant correlations with potential mortality risk, and middle-to-high performance in discriminating mortality events with area under the curve (AUC) values of 0.690, 0.866, 0.833 respectively for independent indicators. The PLR, NLR, and MLR significantly increased within 4 weeks, 1 week, and 48 h before death (*P* < 0.05). As the condition fluctuates, the PLR, NLR, and MLR exhibit fluctuating changes. The NLR was significantly higher in patients with malignant tumors and severe infections compared to those with cardiovascular and cerebrovascular diseases, and the sudden death group (*P* < 0.05).

**Conclusion:**

The PLR, NLR, and MLR significantly associate with mortality risk in hospitalized patients, and could serve as important identification parameters for mortality.

## Introduction

The timely and accurate prediction of mortality risk in hospitalized patients remains a critical challenge in clinical practice. Prognostic evaluation is particularly vital for critically ill individuals, as it guides treatment planning and enhances survival rates (Bauer et al., 2022; Kesecioglu et al., 2024). Inflammation is a central driver of disease progression and mortality, acting as both a protective mechanism and a potential exacerbator of adverse outcomes when dysregulated (Leńska-Mieciek et al., 2023). In critically ill patients, excessive inflammation often leads to organ dysfunction and death. The platelet-to-lymphocyte ratio (PLR), neutrophil-to-lymphocyte ratio (NLR), and monocyte-to-lymphocyte ratio (MLR) serve as simple indicators of inflammatory status, reflecting the dynamic balance between immunity and inflammation through routine blood tests, and hold significant value in prognostic evaluation across various diseases (Liao et al., 2024; Han et al., 2024; Cai et al., 2024; Karra et al., 2023; Wang J. et al., 2022; Mureşan et al., 2022; Zhang et al., 2021a; Kang et al., 2021). NLR captures the interplay between neutrophils and lymphocytes. Neutrophils, as primary effectors of acute inflammation, clear pathogens and damaged tissue, but their overactivation can trigger tissue damage and amplify inflammatory responses. Lymphocytes, in contrast, play a critical role in immune regulation. Elevated NLR typically indicates heightened inflammation and impaired immune function, a feature strongly associated with poor prognosis in sepsis, cardiovascular diseases, and malignancies (Capone et al., 2018). In sepsis patients, enhanced NLR may reflect an aggressive inflammatory cascade leading to multi-organ failure (Zhang et al., 2024). PLR reflects the interaction between platelets and lymphocytes. Beyond their role in coagulation, platelets contribute to inflammation by releasing inflammatory mediators and promoting leukocyte recruitment. Elevated PLR may signal systemic inflammation and increased thrombotic risk, particularly evident in cancer and heart failure diseases (Zhou et al., 2022; Balta and Ozturk, 2015). In cancer, platelets can support tumor growth and metastasis, while in heart failure, the dual effects of inflammation and thrombosis exacerbate mortality risk (Zhou et al., 2022). MLR represents the ratio of monocytes to lymphocytes. Monocytes, key players in innate immunity, differentiate into macrophages and dendritic cells (DCs), regulating inflammation and tissue repair. Elevated MLR may indicate enhanced monocyte activity or lymphocyte depletion, commonly observed in infectious and chronic inflammatory conditions, and is associated with disease severity and mortality risk (Cheng et al., 2023; Hua et al., 2023).

Mechanistically, PLR, NLR, and MLR may predict mortality by capturing the body’s reaction to pathological stressors such as infection, tissue injury, or chronic disease burden. In acute conditions like sepsis, elevated NLR and MLR often correlate with non-survival, likely due to uncontrolled inflammation leading to organ failure (Li et al., 2024). In chronic diseases such as cancer, persistent inflammation may induce immune exhaustion, manifested by lymphocyte depletion and elevated PLR, NLR, and MLR, signaling disease progression and increased mortality risk (Cheng et al., 2023; Hua et al., 2023). Additionally, elevated PLR may further influence prognosis by indicating thrombotic risk, such as in cardiovascular events. While sophisticated biomarkers such as procalcitonin, C-reactive protein panels, and interleukin-6 assays provide enhanced identification accuracy for intensive care unit (ICU) mortality risk, their implementation is hindered by high costs (>$50/test) and dependency on advanced laboratory infrastructure, limiting utility in resource-poor settings. In contrast, PLR, NLR, and MLR calculated from standard complete blood counts (CBCs) offer a pragmatic alternative: inexpensive, immediately accessible, and validated across diverse ICU populations with robust associations to outcomes. This selection could be highly recommended in low- and middle-income countries for equitable diagnosis, balancing accuracy with feasibility to inform timely interventions where advanced testing is unavailable.

Although using PLR, NLR, and MLR in identifying mortality risk has been investigated across various conditions, such as sepsis, cancer, and cardiovascular diseases, their dynamic changes in the terminal phase of hospitalized patients remain inadequately characterized. Most studies focus on static measurements at specific time points or in disease-specific cohorts, with limited exploration of longitudinal trends in critically ill patients approaching death. Furthermore, comparisons with powered control groups, such as patients with chronic conditions like kidney disease, rehabilitation patients, or healthy individuals, are scarce, limiting the understanding of these biomarkers’ specificity in mortality prediction. This study would address these gaps by conducting a large-scale retrospective analysis of PLR, NLR, and MLR trajectories prior to death in hospitalized patients, compared with kidney disease cases, rehabilitation patients, and healthy controls. By elucidating their temporal dynamics, association with mortality events and applicability in discriminating mortality risk, this study aimed to expand these indicators’ clinical utility and provide novel insights into mortality risk evaluation.

## Materials and methods

### General data

We conducted a retrospective cohort study of 832 hospitalized patients who died at Changzhou De’an Hospital between January 2016 and October 2020. Complete medical records and laboratory results were available for 341 patients, who were included in the mortality group. This group comprised 192 males (age range: 32–97 years; mean age: 73.66 ± 13.75 years) and 149 females (age range: 30–98 years; mean age: 79.82 ± 13.46 years). Inclusion criteria were as follows: (1) death during hospitalization; (2) availability of complete medical records; (3) blood routine test results available within 48 h prior to death; and (4) no history of hematological disorders. Exclusion criteria included: (1) fewer than three blood routine tests during hospitalization; (2) presence of hematological conditions that could confound results; and (3) use of medications known to alter platelet or white blood cell counts. Baseline data, including age, sex, laboratory results, medical history, and comorbidities, were extracted from patient records. Additionally, 210 patients with kidney disease and 210 rehabilitation patients, hospitalized during the same period, served as disease control groups, while 689 hospital staff members constituted the healthy control group. Patients with chronic kidney disease (CKD) were allowed to be included into the kidney disease group. In this case, CKD is defined as the presence of renal injury or a decrease of at least 3 months in the glomerular filtration rate (GFR) below 60 mL/min/1.73 m^2^. According to GFR, CKD could be classified into five stages and the final stage of CKD is defined in the condition that GFR <15 mL/min/1.73 m^2^ and requires renal replacement treatment (Jha et al., 2013). Many factors such as hypertension, dyslipidemia, diabetes, aging and gender are involved in the degradation of renal function and implicitly in the evolution of CKD (Vivante, 2024; Vivante and Hildebrandt, 2016). Associations between inflammatory markers and CKD have been studied recently, NLR serves as a predictive role in cardiovascular disease (CVD) while its association to CKD stays in the initial investigation stage (Snoek et al., 2022; Khan et al., 2022). The rehabilitation group involves individuals who ever stayed in ICU and got rehabilitation care after leaving ICU. There are growing body of evidence evaluating rehabilitation interventions after discharge from hospital, while studies evaluating the effects of rehabilitation on survivors with critical illness after discharge from ICU are limited (Connolly et al., 2015). We notice the number of post-ICU clinics is emerging since COVID-19 pandemic where there are ICU experts, rehabilitation specialists, psychologists, pharmacists, nurses, and social workers (Schofield-Robinson et al., 2018; Hiser et al., 2025). However, evidence supporting the efficacy of post-ICU clinics on long term muscle weakness and physical functioning is unclear.

### Blood sample collection and analysis

Skilled and authorized nurses in our hospital collected venous blood from the participants, the participants’ venous blood samples were gathered using K2 EDTA tubes and underwent analysis within a timeframe of 2 h. NLR, PLR and MLR was calculated derived from CBC per individual. Complete blood count was determined by Sysmex XN2800 (Kobe, Japan). The venous blood samples of participants were taken into sterile drying tubes, and the serum taken after centrifugation within 2 h. All blood samples were taken within 2 h. Using the Beckman Coulter LH750 fully automated blood cell analyzer and its compatible reagents, multiple routine blood samples were collected and the most recent blood sample near to patient death was analyzed in current study to calculate the PLR, NLR, and MLR. For the blood samples from individuals in kidney disease group, rehabilitation group and healthy control group, as there was no death, the latest blood sample accessible to us was used and analyzed for PLR, NLR, and MLR. Beyond, other blood biochemistry results for participants in mortality group were obtained without receiving any medications that could alter platelet and WBC. PLR was calculated via platelet count divided by lymphocyte count, NLR was calculated via neutrophil count divided by lymphocyte count, MLR was calculated by monocyte count divided by lymphocyte count. Furthermore, all tests were carried out in strict accordance with the regulations and tested by professional laboratory technician. The instruments are in control during the testing period.

### Statistical analysis

Descriptive statistics were reported as means ± standard deviations (SD) or medians with interquartile ranges (IQR) for continuous variables, and as frequencies with percentages for categorical variables. Group differences were analyzed using chi-square tests (or Fisher’s exact test) for categorical variables and one-way ANOVA or the Kruskal–Wallis *H* test for continuous variables, as appropriate.

Since individuals in mortality group, kidney disease group, rehabilitation group and healthy group could be regarded as suffered different physical/health conditions like death, kidney disease, in rehabilitation and healthy conditions, we aimed to investigate the association between different PLR, NLR and MLR values and those physical conditions. Multivariable logistic regression models were used to estimate odds ratios (ORs) with 95% confidence intervals (CIs) for the risk of death vs. kidney disease, death vs. rehabilitation and death vs. healthy conditions, estimates were adjusted for age, gender, body mass index (BMI) hemoglobin, total cholesterol (TC) and triglyceride (TG), history of cancer, history of CVD, history of diabetes and history of neurological disease to provide robust results (Ben Salem and Ben Abdelaziz, 2020). Considering potential heterogeneity among the mortality group, kidney disease group, rehabilitation group and healthy control group, we performed propensity score matching (PSM) analysis on baseline features of age, gender and BMI to make these groups comparable with less heterogeneity as kind of sensitivity analysis, also we repeated the multivariable logistic regression analysis based on the matched populations after PSM to demonstrate the consistency and robustness. Besides, the receiver operating characteristic (ROC) curve was used to evaluate the efficacy of PLR, NLR, and MLR in predicting mortality risk, including the calculation of the area under the curve (AUC), optimal cutoff values, sensitivity, specificity, and Youden’s index. We used the Youden’s index defined as J = Sensitivity + Specificity – 1 to determine the optimal cut-off value by identifying the threshold that maximizes this index on the ROC curve (Hughes, 2015). In the ROC curve, the cut-off value represented the probability threshold that best balanced true positive rate (sensitivity) and false positive rate (1-specificity) for classifying predictions. This optimal cut-off helped to achieve the best trade-off between correctly identifying positive cases and minimizing false positives in a binary classification model. The correlation of non-normally distributed data was assessed using Spearman’s rank correlation to compare correlations between different groups. Statistical analyses were performed using SPSS version 25.0 to analyze the collected clinical data and test results. A *P*-value less than 0.05 was considered statistically significant. Ethical approval was granted by De’an Hospital with informed consent obtained from all participants in mortality group, kidney disease group and rehabilitation group. Application for data use in healthy control group was reviewed and secured by De’an Hospital.

## Results

### Baseline characteristics

**FIGURE 1:**
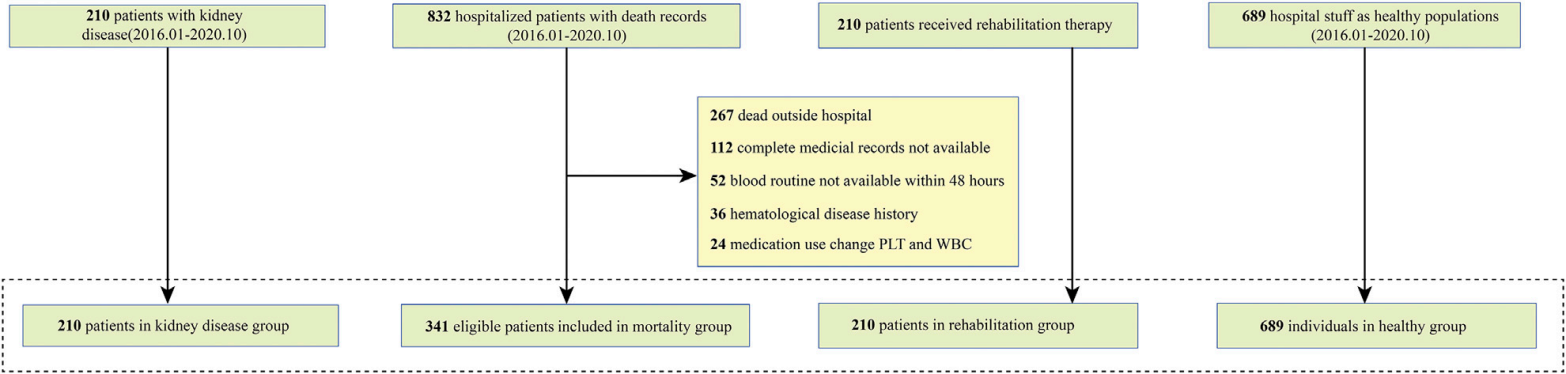
The flow chart for included participants in four groups.

Following the selection criteria, there were 832 hospitalized participants with confirmed death records between 2016.01 and 2020.10 reviewed. After removing the ineligible participants who were mostly dead outside the hospital or could not provide complete medical records or could not provide timely blood routine test results, 341 patients were eligibly included in the mortality group. Besides, we selected 210 kidney disease patients and 210 patients receiving rehabilitation management in kidney disease group and rehabilitation group respectively. To extend the utility of our findings, we also included 689 hospital staff as volunteers in the healthy control group (Figure 1).

Demographic characteristics were recorded in Table 1, the mean age in mortality group was 75.86 ± 11.75 years, in kidney disease group was 52.16 ± 12.46 years, and were 58.22 ± 10.32 years and 43.12 ± 12.67 years in rehabilitation group and healthy group respectively. For some common plasma biochemistry results, healthy group individuals showed significantly better profiles over the other three groups which were mostly comparable except uric acid and serum creatinine, also the mortality group individuals possessed higher C-reactive protein (CRP). Similarly, for comorbidities and life habit history (like drinking and smoking history), healthy group populations also suffered less than other groups. For the death reasons of individuals in mortality group, 35.2% and 21.4% individuals died due to CVD and cancer, 15.2% and 15.2% patients died for infectious disease and multiple organ dysfunction syndrome (MODS), 9.7% patients were due to neurological/psychiatric diseases, only 3.2% populations died for trauma.

**TABLE 1:** Baseline characteristics of included participants in different groups.

	Mortality group (n = 341) (n±SD/n(%))	Kidney disease group (n = 210) (n±SD/n(%))	Rehabilitation group (n = 210) (n±SD/n(%))	Healthy group (n = 689) (n±SD/n(%))
Age (years)	75.86 ± 11.75	52.16 ± 12.46	58.22 ± 10.32	43.12 ± 12.67
Men (n, %)	192 (56.3)	151 (71.9)	127 (60.5)	361 (52.4)
BMI	19.64 ± 3.26	20.14 ± 2.12	20.62 ± 2.32	22.45 ± 2.65
History of cancer	92 (27.0)	15 (7.1)	20 (9.5)	12 (1.7)
History of CVD	108 (31.7)	72 (34.3)	79 (37.6)	26 (3.8)
History of diabetes	86 (25.2)	69 (32.9)	59 (28.1)	22 (3.2)
History of neurological disease	44 (12.9)	24 (11.4)	27 (12.9)	56 (8.1)
History of drinking	249 (73.0)	158 (75.2)	154 (73.3)	311 (45.1)
History of smoking	116 (34.0)	108 (51.4)	113 (53.9)	150 (21.8)
Hemoglobin (g/L)	121.56 ± 20.37	124.74 ± 18.64	126.62 ± 13.24	138.12 ± 14.91
Serum albumin (g/L)	37.26 ± 5.54	34.47 ± 3.66	37.27 ± 6.82	45.14 ± 5.01
Uric acid (μmol/L)	429.56 ± 92.64	482.16 ± 105.33	416.22 ± 60.64	311.22 ± 65.27
Serum creatinine (μmol/L)	96.67 ± 28.14	134.77 ± 40.25	84.65 ± 30.24	75.12 ± 33.54
Total cholesterol (mmol/L)	4.25 ± 1.75	4.84 ± 2.32	4.64 ± 1.44	4.23 ± 1.24
Triglyceride (mmol/L)	1.18 ± 1.19	1.42 ± 1.25	1.38 ± 1.12	1.34 ± 1.83
HDL-C (mmol/L)	1.08 ± 0.65	1.32 ± 1.14	1.31 ± 0.84	1.36 ± 0.99
LDL-C (mmol/L)	2.18 ± 1.02	2.86 ± 0.40	2.76 ± 1.42	2.42 ± 1.21
CRP (mg/L)	4.84 ± 0.98	2.36 ± 1.53	2.96 ± 1.03	1.10 ± 0.86
Death reasons				
Cancer	73 (21.4)	0	0	0
Infectious disease	52 (15.2)	0	0	0
CVD	120 (35.2)	0	0	0
Neurological and psychiatric diseases	33 (9.7)	0	0	0
MODS	52 (15.2)	0	0	0
Trauma	11 (3.2)	0	0	0

Abbreviations: SD, stand deviation; BMI, body mass index; CVD, cardiovascular disease; HDL-C, high-density lipoprotein cholesterol; LDL-C, low-density lipoprotein cholesterol; CRP, C-reactive protein; MODS, multiple organ dysfunction syndrome.

After PSM, there were 129 individuals in mortality group, kidney disease group, rehabilitation group and healthy control group respectively, then we observed there were no significant differences on age, gender and BMI. Besides, other covariables kept consistent trends with the demographic distribution before PSM (Supplementary Table S1).

### PLR, NLR, and MLR values among mortality, rehabilitation and healthy control groups

The mortality group exhibited a median PLR of 166.04, significantly higher than that of the kidney disease group (131.39), rehabilitation group (130.63), and healthy control group (100.92). Similarly, the median NLR in the mortality group was 5.69, compared to 3.10 in the kidney disease group, 2.41 in the rehabilitation group, and 1.57 in the healthy control group. The median MLR was 0.49 in the mortality group, notably elevated relative to 0.32 in the kidney disease group, 0.28 in the rehabilitation group, and 0.18 in the healthy control group. Kruskal–Wallis H tests confirmed significant differences in PLR, NLR, and MLR across the mortality, kidney disease, rehabilitation, and healthy control groups, yielding H values of 244.086, 683.779, and 598.623, respectively (all *P* < 0.001). Post-hoc pairwise comparisons using the Bonferroni correction revealed significant differences in PLR, NLR, and MLR between the mortality group and each control group (adjusted *P* < 0.001). However, no significant differences were observed in PLR or MLR between the kidney disease and rehabilitation groups (adjusted *P* = 1.000 and 0.132, respectively). Detailed results are presented in Table 2.

**TABLE 2:** Comparison of PLR, NLR, and MLR among different patient groups.

Category	PLR median, (IQR)	NLR median, (IQR)	MLR median, (IQR)
Mortality group(n = 341)	166.04(103.02,263.80)*	5.69(3.24,9.77)*	0.49(0.32,0.79)*
Kidney disease group(n = 210)	131.39(101.33,184.88)	3.10(2.37,4.69)	0.32(0.24,0.42)
Rehabilitation group(n = 210)	130.63(103.18,175.17)	2.41(1.79,3.51)	0.28(0.21,0.38)
Healthy group(n = 689)	100.92(82.49,125.19)	1.57(1.29,2.02)	0.18(0.15,0.23)
H-value	244.086	683.779	598.623
*P*-value	0.000	0.000	0.000

### Comparison of PLR, NLR, and MLR in deceased hospitalized patients by age and gender

This study stratified deceased hospitalized patients by gender into two groups: 192 males and 149 females. Median values of PLR, NLR, and MLR were compared between the groups. Among male patients, the median PLR, NLR, and MLR were 168.25, 5.90, and 0.51, respectively, whereas among female patients, the corresponding values were 164.00, 5.10, and 0.45. The Mann–Whitney U test indicated no statistically significant differences between the sexes, with all *P*-values exceeding 0.05. Patients were further categorized by age into two groups: those younger than 60 years (n = 56) and those aged 60 years or older (n = 285). Median PLR, NLR, and MLR values for the younger group were 135.57, 6.11, and 0.48, respectively, while those for the older group were 170.64, 5.46, and 0.49. The Mann–Whitney U test demonstrated that the PLR was significantly higher in the ≥60 age group (*P* = 0.05), suggesting a potential age-related elevation. However, differences in NLR and MLR between age groups did not reach statistical significance. Detailed results are presented in Table 3.

**TABLE 3:** Comparative analysis of PLR, NLR, and MLR in terminal patients grouped by gender and age.

Category	PLR median, (IQR)	NLR median, (IQR)	MLR median, (IQR)
Gender			
Male(n = 192)	168.25(106.29,272.83)	5.90(3.50,10.40)	0.51(0.33,0.79)
Female(n = 149)	164.00(96.58,255.24)	5.10(2.71,9.33)	0.45(0.29,0.74)
Z-value	−0.727	−1.844	−1.27
*P*-value	0.467	0.065	0.201
Age			
≤60(n = 56)	135.57(86.59,236.34)	6.11(3.34,8.30)	0.48(0.32,0.74)
>60(n = 285)	170.64(105.51,277.77)	5.46(3.17,10.03)	0.49(0.32,0.79)
Z-value	−1.959	−0.047	−0.421
*P*-value	0.05	0.962	0.674

### The association between PLR, NLR and MLR and mortality risk

We performed binary multivariable logistic regression analysis evaluating the association between PLR, NLR and MLR and mortality risk comparing individuals in mortality group to kidney disease group, to rehabilitation group, to healthy group respectively (Table 4). After adjusted for age, gender, BMI, hemoglobin, TC, TG and comorbidities, higher PLR, NLR and MLR were all significantly associated with increased mortality risk when comparing to patients in kidney disease group (OR: 1.60, 95% CI: 1.24-2.06, *p* < 0.001 for PLR; OR: 1.70, 95% CI: 1.32-2.19, *p* < 0.001 for NLR; OR: 1.57, 95% CI: 1.22-2.02, *p* < 0.001 for MLR). Comparing to individuals in rehabilitation group, we observed a significantly increased risk of mortality with increased PLR (OR: 1.79, 95% CI: 1.36-2.35, *p* < 0.001), NLR (OR: 1.98, 95% CI: 1.47-2.65, *p* < 0.001) and MLR (OR: 2.18, 95% CI: 1.59-2.98, *p* < 0.001). Consistently, significant association was identified between increased PLR (OR: 2.05, 95% CI: 1.50-2.81, *p* < 0.001), NLR (OR: 2.10, 95% CI: 1.53-2.87, *p* < 0.001) and MLR (OR: 2.46, 95% CI: 1.76-3.43, *p* < 0.001) and enhanced mortality risk when comparing to healthy individuals. These results addressed higher PLR, NLR and MLR value would associate with increased mortality risk.

**TABLE 4:** Multivariable logistic regression analysis results.

Marker	β	SE	Wald	Or (95% CI)^a^	P-value
Mortality group vs. kidney disease group					
PLR	0.470	0.130	13.07	1.60 (1.24–2.06)	<0.001
NLR	0.530	0.130	16.62	1.70 (1.32–2.19)	<0.001
MLR	0.450	0.130	12.00	1.57 (1.22–2.02)	<0.001
Mortality group vs. rehabilitation group					
PLR	0.580	0.140	17.18	1.79 (1.36–2.35)	<0.001
NLR	0.680	0.150	20.53	1.98 (1.47–2.65)	<0.001
MLR	0.780	0.160	23.74	2.18 (1.59–2.98)	<0.001
Mortality group vs. healthy group					
PLR	0.720	0.160	20.25	2.05 (1.50–2.81)	<0.001
NLR	0.740	0.160	21.41	2.10 (1.53–2.87)	<0.001
MLR	0.900	0.170	28.02	2.46 (1.76–3.43)	<0.001

^a^ Logistic regression analysis was adjusted for age, gender, BMI, hemoglobin, total cholesterol, triglyceride and comorbidities including history of cancer, history of CVD, history of diabetes, history of neurological disease.

**Abbreviations**: PLR, platelet-to-lymphocyte ratio; NLR, neutrophil-to-lymphocyte ratio; MLR, monocyte-to-lymphocyte ratio; OR, odd ratio; 95% CI, 95% confidence interval; BMI, body mass index; CVD, cardiovascular disease.

Then in populations after PSM, the results were in line with those without PSM adjustment. In each group with 129 individuals, PLR, NLR and MLR associated with more mortality possibility over patients with kidney disease (OR: 1.52, 95% CI: 1.16-1.99 for PLR; OR: 1.62, 95% CI: 1.23-2.13 for NLR; OR: 1.49, 95% CI: 1.15-1.93 for MLR), over populations had received rehabilitation (OR: 1.68, 95% CI: 1.26-2.24 for PLR; OR: 1.86, 95% CI: 1.36-2.54 for NLR; OR: 2.05, 95% CI: 1.48-2.84 for MLR) and over healthy control individuals (OR: 1.93, 95% CI: 1.42-2.63 for PLR; OR: 1.97, 95% CI: 1.43-2.72 for NLR; OR: 2.32, 95% CI: 1.65-3.26 for MLR) (Supplementary Table S2). Before and after PSM, our results exhibited good homogeneity.

### Performance of PLR, NLR, and MLR in identifying mortality risk

**FIGURE 2:**
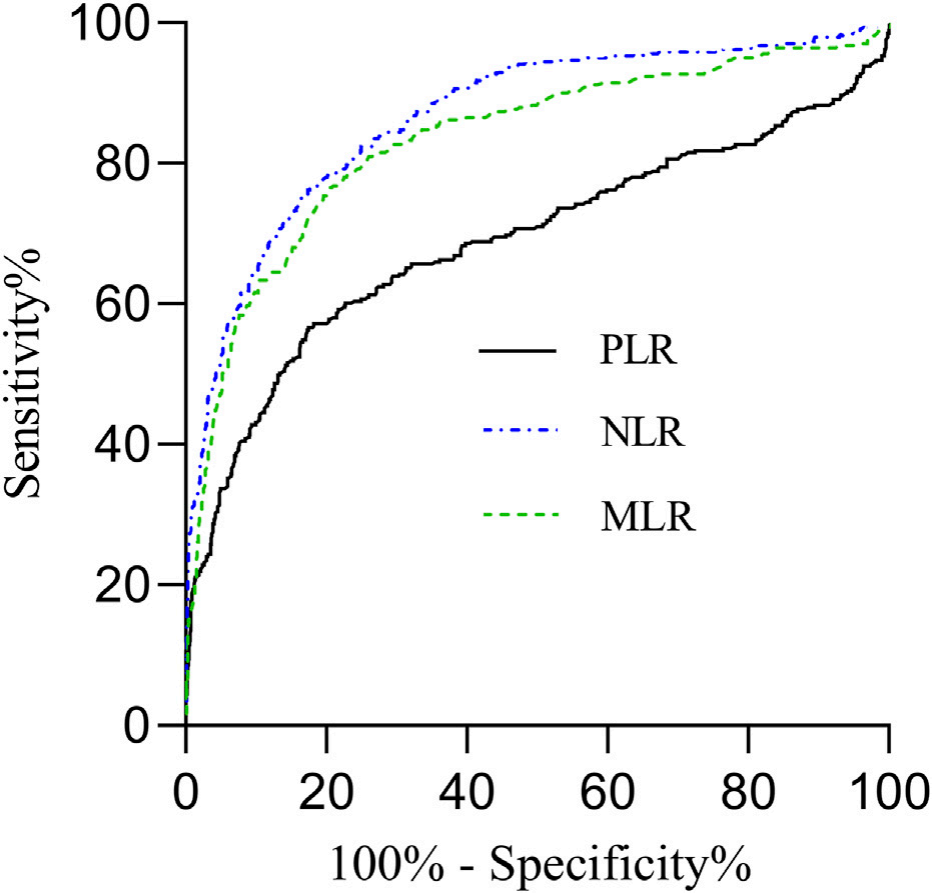
ROC curves for identifying mortality risk in hospitalized patients using PLR, NLR, MLR.

We found the performance of PLR, NLR, and MLR significantly differ in the identification on mortality risk among hospitalized patients when compared with renal, rehabilitative, and healthy control groups (*P* < 0.05). As showed in ROC curves, the AUC for NLR is 0.866, with an optimal cut-off value of 3.79, yielding a sensitivity of 0.674 and a specificity of 0.890. MLR demonstrates an AUC of 0.833, with a cut-off value of 0.31, sensitivity of 0.768, and specificity of 0.791. PLR exhibits lower identification power, with an AUC of 0.690, a threshold of 154.28, sensitivity of 0.566, and specificity of 0.825. These ROC results are summarized in Table 5 and illustrated in Figure 2.

**TABLE 5:** The predictive value of combined PLR, NLR, and MLR for mortality in hospitalized patients.

Parameter	Optimal cut-off value	AUC	Standard Error	95%CI	*P*-value	Sensitivity	Specificity	Youden index
PLR	154.28	0.690	0.019	0.654–0.73	0.000	0.566	0.825	0.391
NLR	3.79	0.866	0.012	0.843–0.89	0.000	0.674	0.890	0.564
MLR	0.31	0.833	0.014	0.806–0.861	0.000	0.768	0.791	0.559

### Analysis of PLR, NLR, and MLR values at different time points prior to death

**FIGURE 3:**
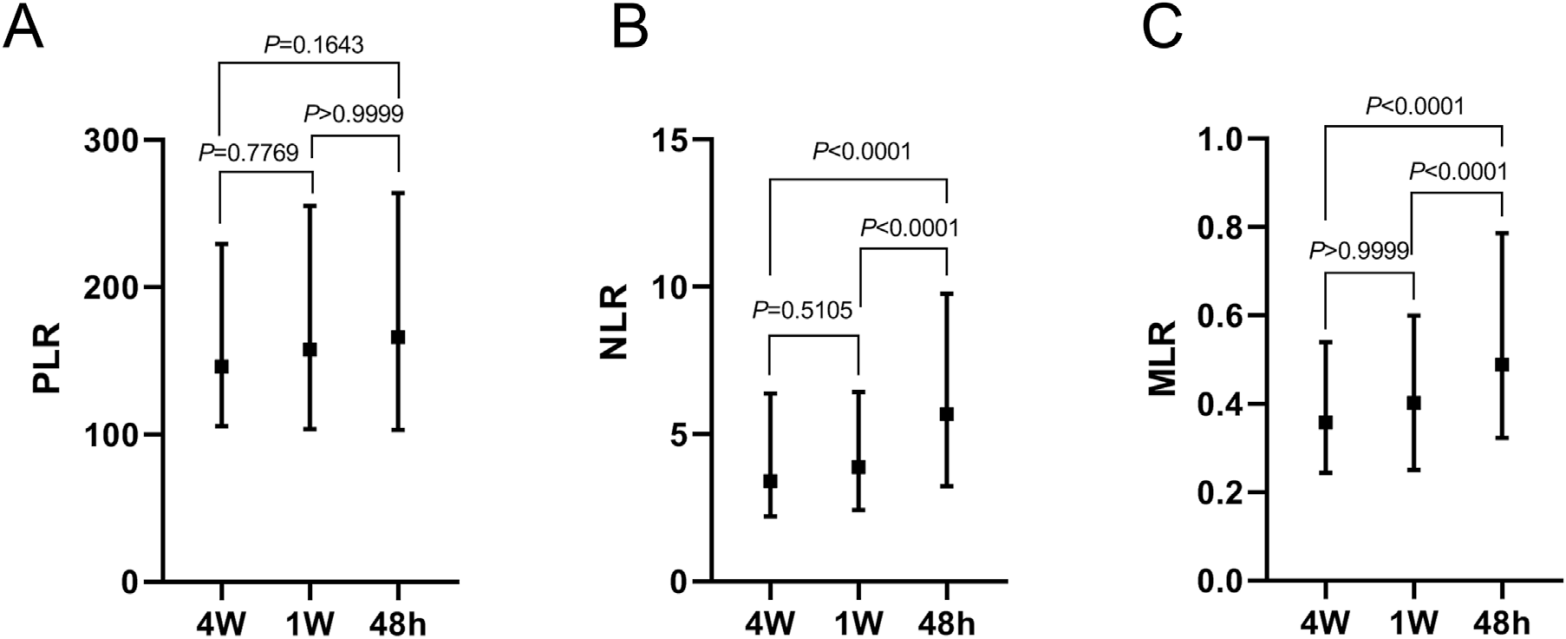
**(A)** Comparative analysis of median PLR measurements from the three tests conducted prior to death; **(B)** Comparative analysis of median NLR measurements from the three tests conducted prior to death; **(C)** Comparative analysis of median MLR measurements from the three tests conducted prior to death.

At 4 weeks prior to death, the median values of PLR, NLR, and MLR were 146.09, 3.41, and 0.36, respectively. One week before death, these values increased to 157.56 for PLR, 3.89 for NLR, and 0.40 for MLR. Approximately 48 h prior to death, the final recorded values were 166.04 for PLR, 5.69 for NLR, and 0.49 for MLR. Although PLR showed a gradual increase over the three time points, the changes were not statistically significant (*P* > 0.05). Similarly, NLR and MLR values exhibited slight increases between the 4-week and 1-week time points, which were also not statistically significant (*P* > 0.05). However, measurements taken approximately 48 h prior to death were significantly elevated compared to those at the earlier time points (*P* < 0.001). These trends are illustrated in Figure 3.

### Analysis of PLR, NLR, and MLR results at different treatment stages preceding death

Among 63 patients who underwent multiple (≥4) blood tests prior to death, PLR, NLR, and MLR levels exhibited wave-like fluctuations. These biomarkers were significantly elevated during episodes of clinical deterioration and at the end-of-life stage compared to their levels at hospital admission and during periods of remission, with statistically significant differences observed. Detailed results are presented in Table 6.

**TABLE 6:** Comparison of median PLR, NLR, and MLR values across different treatment periods.

Category	PLR median, (IQR)	NLR median, (IQR)	MLR median, (IQR)
Admission	148.60(110.98,220.00)	3.25(2.17,5.27)	0.32(0.23,0.48)
Deterioration period	246.05(169.40,347.67)*	8.39(5.33,11.46)*	0.58(0.42,0.75)*
Remission period	144.44(104.89,202.70)	3.17(2.25,3.84)	0.33(0.27,0.44)
Terminal phase	250.45(171.29,368.69)**	7.83(5.05,16.96)**	0.69(0.41,0.95)**
H-value	48.96	104.30	63.17
*P*-value	0.000	0.000	0.000

* Significantly different from admission and remission periods, with *P* < 0.001.

** Significantly different from admission and remission periods, with *P* < 0.001.

### Correlation analysis of PLR, NLR, and MLR in mortality risk prediction

**FIGURE 4:**
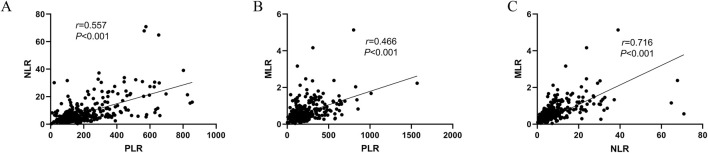
**(A)** Correlation chart for PLR and NLR; **(B)** Correlation chart for PLR and MLR; **(C)** Correlation chart for NLR and MLR.

Spearman’s correlation analysis demonstrated moderate to strong positive correlations among the three inflammatory markers. Specifically, the correlation coefficient between PLR and NLR was 0.557, between PLR and MLR was 0.466, and between NLR and MLR was 0.716. All correlations were statistically significant (*P* < 0.001) (Figure 4).

### Analysis of PLR, NLR, and MLR in relation to different causes of death

A total of 341 deceased patients were classified into six groups according to the cause of death: malignancy, severe infection, cardiovascular and cerebrovascular diseases, multiple organ failure, severe trauma, and sudden death. The median values of PLR, NLR, and MLR were calculated and compared across these groups. Notably, NLR levels in the malignancy and severe infection groups were significantly higher than those in the cardiovascular and cerebrovascular diseases group and the sudden death group (*P* < 0.01). In contrast, differences in PLR and MLR values among the groups were not statistically significant. Detailed results are presented in Table 7.

**TABLE 7:** Analysis of median values of PLR, NLR, and MLR according to varying causes of death.

Category	PLR median, (IQR)	NLR median, (IQR)	MLR median, (IQR)
Malignancy(n = 73)	157.23(93.60,344.28)	7.25(5.01,11.86)*	0.54(0.37,0.82)
Severe infection(n = 52)	208.63(135.73,277.53)	6.86(3.97,13.21)*	0.47(0.30,0.86)
Cardiovascular and cerebrovascular diseases(n = 120)	172.25(104.62,253.23)	5.07(2.71,8.91)**	0.47(0.33,0.80)
Multiple organ failure(n = 52)	147.70(89.82,238.24)	5.06(2.85,8.65)	0.53(0.34,0.76)
Severe trauma(n = 10)	182.93(132.35,333.52)	7.91(4.19,10.46)	0.45(0.41,0.76)
Sudden death(n = 33)	123.68(85.09,231.19)	3.34(2.43,5.72)	0.35(0.26,0.55)
H-value	7.558	25.229	8.393
*P*-value	0.182	0.000	0.136

## Discussion

Accurate and timely identification of inpatient mortality risk is essential for enabling early intervention and optimizing treatment strategies, thereby advancing personalized medicine. Inflammatory responses significantly influence disease progression and prognosis, with a range of biomarkers available to monitor these processes. Among these, the PLR, NLR, and MLR have emerged as novel inflammatory markers. These ratios are increasingly utilized to assess inflammatory responses and immune status, playing a critical role in disease diagnosis, condition monitoring, and risk evaluation (Kang et al., 2021; Qiu et al., 2023; Tudurachi et al., 2023; Duman et al., 2021; Kulakli et al., 2023; Shen et al., 2017; Song et al., 2022; Lee and Song, 2024).

In this study, we found that the median levels of PLR, NLR, and MLR prior to patient demise were significantly higher than those in the kidney disease, rehabilitation, and healthy control groups. This observation suggests that elevated PLR, NLR, and MLR levels are associated with increased mortality risk in hospitalized patients. The rise in these markers likely reflects an intensified inflammatory state coupled with suppressed immune function, both characteristic of critical illness. Platelets contribute to inflammation beyond their role in coagulation, while lymphocytes regulate immune responses. Consequently, an elevated PLR may indicate both heightened inflammation and immune suppression. Previous studies have reported a progressive decline in platelet counts toward the end of life, with the increase in PLR primarily driven by reduced lymphocyte counts (Zhang et al., 2014). This pattern aligns with our findings and emphasizes the importance of lymphocyte dynamics in critically ill patients. Similarly, PLR has been closely linked to disease severity and prognosis in conditions such as cancer and autoimmune disorders (Smith et al., 2009; Adhyatma and Warli, 2019), reinforcing its prognostic utility across diverse contexts.

Neutrophils, as the body’s first responders to acute inflammation, exhibited a significant increase before death, reflecting a stress response to severe disease states. Concurrently, a relative reduction in lymphocyte counts suggests immune system failure. The NLR, a widely recognized marker of inflammation and immune activity, has been shown to outperform traditional inflammatory biomarkers such as C-reactive protein in various settings (Li et al., 2020; Lorente et al., 2022). Its elevation is consistently associated with poor prognosis in cardiovascular diseases, malignancies, and infectious conditions (Asik, 2021; Yoon and Lee, 2021). For example, Chen et al. reported that elderly patients with acute myocardial infarction and high NLR faced a 3.091-fold increased risk of in-hospital mortality (Chen et al., 2023), a finding that parallels our results across a broader hospitalized population. In contrast, some studies report lower NLR thresholds in specific diseases; for instance, Zahorec et al. suggested that NLR values above 4.0 indicate severe stress in critical care settings (Zahorec, 2021), highlighting potential variability influenced by patient cohorts or disease states.

Monocytes, key players in immune and inflammatory responses, also contribute to mortality prediction. An elevated MLR suggests immune suppression and activation of inflammatory pathways. In malignancies, increased MLR has been associated with tumor immune evasion, possibly driven by the inflammatory microenvironment (Li et al., 2023; Jakubowska et al., 2020; Wang Q. et al., 2022). Our study observed higher MLR levels in patients with malignant tumors, consistent with reports by Wang et al., who linked elevated MLR to worse survival in lung cancer patients (Wang J. et al., 2022). However, discrepancies exist; unlike NLR, MLR’s prognostic value appears less consistent across non-malignant conditions, potentially due to differences in monocyte activation patterns (Citu et al., 2022). This variability underscores the need to contextualize MLR findings within specific disease frameworks.

Our analysis revealed a trend toward higher PLR, NLR, and MLR levels in male patients compared to females, though this difference was not statistically significant. This observation may stem from gender-based differences in hormone levels, immune responses, or physiological functions. Estrogen, known to modulate immune activity, may dampen inflammatory responses in females, as supported by studies on sex-specific immune profiles (Davis and Baber, 2022). Additionally, PLR was significantly elevated in patients over 60 years, suggesting greater sensitivity in older populations. This age-related trend aligns with findings by Duan et al., who noted that PLR’s prognostic accuracy increases with age due to cumulative inflammatory burden (Duan et al., 2023).

A distinctive feature of this study is we achieved PLR, NLR and MLR at multiple time points before death. Four weeks prior to demise, median levels of these markers were elevated compared to other disease groups, though not significantly. This subtle rise may reflect early physiological changes preceding clinical decline. By 1 week before death, the markers increased further, and within the final 48 h, a sharp escalation occurred, likely indicating severe inflammation and immune dysfunction (Wang et al., 2023). This temporal pattern is consistent with palliative care research, where inflammatory markers surge as death nears (Suh et al., 2019). Compared to cross-sectional studies like that of Templeton et al., which focused on single-point NLR measurements in cancer (Templeton et al., 2014), our dynamic approach offers novel insights into biomarker trajectories, enhancing their clinical relevance.

In a subset of 63 hospitalized patients, we observed significant increases in PLR, NLR, and MLR during episodes of infection, exacerbated inflammation, or tumor progression (Zhang et al., 2023). Following anti-inflammatory or targeted treatments, these ratios declined, reflecting control over inflammation. However, this reduction was often transient, with subsequent rises during disease recurrence. This fluctuating pattern mirrors findings by Liu et al., who noted similar biomarker dynamics in sepsis patient post-treatment (Liu et al., 2021). Unlike their study, which primarily focused on NLR, our inclusion of PLR and MLR provides a broader inflammatory profile, potentially enhancing the monitoring of treatment response and disease progression.

ROC curves illustrated middle-to-high discriminating ability for mortality risk, while NLR exhibited the highest AUC, sensitivity, and specificity, positioning its superiority as a good clinical tool. Our NLR cutoff aligns closely with Zhang et al.’s threshold of 3.93 for cerebral venous thrombosis (Zhang et al., 2021b), while our PLR cut-off (154.28) is comparable to their 149.52, suggesting consistency across contexts. Although we did not provide the ROC for the combining PLR, NLR and MLR together to avoid overfitting risk in ROC model, results from Wang et al., demonstrated improved utility in identification of diabetes with combined multi-marker model (Wang et al., 2020). However, we should be cautious to interpret their results.

A significant positive correlation between PLR, NLR, and MLR was observed in patients nearing death, consistent with Wang et al.’s findings in diabetic cohorts (e.g., r = 0.609 for NLR and MLR, *P* < 0.001) (Wang et al., 2020). This correlation likely reflects synchronized activation of platelets, neutrophils, and monocytes during severe inflammation, contributing to cytokine production and immune defense. While PLR and MLR showed relative stability across death categories, NLR was significantly higher in patients with malignant tumors and severe infections compared to cardiovascular or sudden death cases. This aligns with Hirahara et al.’s use of NLR to assess tumor response (Hirahara et al., 2019) and Liang et al.’s findings on its predictive value in sepsis mortality (Liang and Yu, 2022). However, our results differ from those of Forget et al., who reported less pronounced NLR variation in non-infectious conditions (Forget et al., 2017), possibly due to differences in sample size or disease severity.

After comparing to other studies, current study has limitations. First of all, this study is limited by the observational nature which should be highly addressed. Although we performed PSM analysis to make individuals in different groups more comparable, selection and recall biases could not be totally excluded. Then, as a single-center retrospective analysis with a modest sample size, its generalizability is limited. Multi-center studies with larger cohorts are needed to validate these findings. Additionally, confounding factors such as underlying disease heterogeneity, therapeutic interventions, and variability in laboratory equipment may have influenced biomarker levels. Future research should incorporate standardized protocols to mitigate these effects. Thirdly, we evolved biochemistry values in longitudinal manner, but we did not have control groups like the cross-sectional analysis. On one hand, it is quite difficult in clinical practice to achieve the matched control cohorts in different timepoints as expected, on the other hand the shortcoming will relatively impair the translational significance of current results. Thus, we recommend future studies involve PSM principles or target trial emulation (TTE) framework to address this issue and serve as good supplements on current results. TTE represents a robust approach to causal inference in observational studies, emulating the design of a hypothetical randomized controlled trial (RCT) using real-world data to mitigate biases inherent in non-experimental settings (Wang et al., 2025; Lin et al., 2025; Yang et al., 2025). By explicitly defining key trial components such as eligibility criteria, treatment strategies (e.g., interventions targeting PLR/NLR reduction via anti-inflammatory therapies), time zero (baseline assessment), follow-up periods, and outcome ascertainment, TTE ensures that analyses align with the counterfactual framework, thereby enhancing internal validity without requiring prospective randomization. In the context of our study, TTE framework could be applied to simulate a target trial evaluating whether modulating PLR and NLR (as exposures) causally influences mortality risk across patient groups. For instance, using electronic health records or cohort data, we could define interventions versus no interventions with intention-to-treat analyses accounting for time-varying confounders via inverse probability weighting (IPW). The emulation would address potential immortal time bias and time-dependent confounding, which are limitations of our current retrospective design. Beyond TTE, complementary statistical methods for trial emulation include instrumental variable analysis (e.g., leveraging genetic variants as instruments for PLR) and doubly robust estimators, which combine outcome regression with IPW to improve efficiency and robustness against model misspecification (Bidulka et al., 2024; Larsson et al., 2023). These approaches, while computationally intensive, offer avenues for future extensions of our work, particularly in prospective validation studies. Nonetheless, TTE’s structured emulation remains particularly advantageous for modifiable exposures like PLR and NLR, as it facilitates policy-relevant inferences on preventive strategies. Future research should prioritize TTE in longitudinal cohorts to definitively establish causality and guide clinical interventions. Finally, because the kidney disease group, rehabilitation group, and healthy control group were introduced as external controls, potential demographic heterogeneity should be acknowledged (Yang et al., 2024). Although we performed PSM and additional multivariable logistic regression analyses as sensitivity analyses, these approaches reduced, but did not completely remove the heterogeneity and inherent observational biases. To further clarify the sources of heterogeneity, subgroup and stratified analyses would be warranted.

## Conclusion

In conclusion, this study demonstrates that elevated PLR, NLR, and MLR are significantly associated with mortality risk in hospitalized patients. Monitoring of these ratios offers a simple, cost-effective mortality risk assessment framework, providing clinicians with actionable insights. However, their interpretation requires consideration on comparable control groups and multi-center powered sample size, given potential influences from observational nature. Future studies should apply more advanced statistical methods, recruit large-scale participants and elucidate the underlying biological processes driving these biomarker changes.

## Data Availability

The raw data supporting the conclusions of this article will be made available by the authors, without undue reservation.

## Abbreviations

PLR, platelet-to-lymphocyte; NLR, neutrophil-to-lymphocyte ratio; MLR, monocyte-to-lymphocyte ratio; DCs, dendritic cells; ICU, intensive care unit; CBC, complete blood count; CKD, chronic kidney disease; GFR, glomerular filtration rate; CVD, cardiovascular disease; SD, standard deviation; IQR, interquartile ranges; ORs, odds ratios; 95% CI, 95% confidence interval; BMI, body mass index; TC, total cholesterol; TG, triglyceride; PSM, propensity score matching; ROC, receiver operating characteristic; AUC, area under the curve; CRP, C-reactive protein; MODS, multiple organ dysfunction syndrome; TTE, target trial emulation; RCT, randomized controlled trial; IPW, inverse probability weighting.
