# Supplementary material for: The clinical value of PLR, NLR, and MLR in predicting mortality risk in hospitalized patients
Source: Front Pharmacol. 2025 Dec 3;16:1736947. doi: 10.3389/fphar.2025.1736947 (PMC12708535; doi:10.3389/fphar.2025.1736947)
Supplement: Supplementary file 1 [file Table1.docx]

**Supplementary Table 1**. Baseline characteristics of participants after propensity score matching in different groups

|  | **Mortality group (n=129)**  **(n±SD/n(%))** | **Kidney disease group (n=129) (n±SD/n(%))** | **Rehabilitation group (n=129) (n±SD/n(%))** | **Healthy group (n=129) (n±SD/n(%))** |
| --- | --- | --- | --- | --- |
| Age (years) | 58.43 ± 10.91 | 58.22 ± 12.46 | 58.37 ± 10.32 | 58.12 ± 11.24 |
| Men (n, %) | 78 (60.5) | 79 (61.2) | 78 (60.5) | 77 (59.7) |
| BMI | 20.61 ± 2.52 | 20.14 ± 2.21 | 20.64 ± 2.32 | 20.37 ± 2.46 |
| History of cancer | 26 (20.2) | 11 (8.5) | 20 (15.5) | 12 (9.3) |
| History of CVD | 39 (30.2) | 41 (31.8) | 45 (34.9) | 7 (5.4) |
| History of diabetes | 29 (22.5) | 38 (29.5) | 35 (27.1) | 5 (3.9) |
| History of neurological disease | 19 (14.7) | 9 (7.0) | 14 (11.8) | 2 (1.6) |
| History of drinking | 80 (62.0) | 88 (68.2) | 84 (65.1) | 49 (38.0) |
| History of smoking | 39 (30.2) | 56 (43.4) | 60 (46.5) | 20 (15.5) |
| Hemoglobin (g/L) | 123.15 ± 19.84 | 126.27 ± 16.74 | 128.72 ± 14.61 | 136.35 ± 15.59 |
| Serum albumin (g/L) | 37.52 ± 5.24 | 35.24 ± 3.82 | 37.91 ± 6.44 | 44.81 ± 4.77 |
| Uric acid (μmol/L) | 430.95 ± 92.14 | 485.63 ± 103.64 | 415.61 ± 61.27 | 312.24 ± 66.45 |
| Serum creatinine (μmol/L) | 98.24 ± 29.41 | 136.47 ± 41.36 | 85.42 ± 27.13 | 74.05 ± 32.36 |
| Total cholesterol (mmol/L) | 4.30 ± 1.54 | 4.82 ± 2.30 | 4.60 ± 1.43 | 4.25 ± 1.20 |
| Triglyceride (mmol/L) | 1.20 ± 1.10 | 1.40 ± 1.22 | 1.36 ± 1.08 | 1.30 ± 1.75 |
| HDL-C (mmol/L) | 1.08 ± 0.62 | 1.30 ± 1.10 | 1.33 ± 0.82 | 1.37 ± 0.65 |
| LDL-C (mmol/L) | 2.20 ± 1.00 | 2.85 ± 0.38 | 2.74 ± 1.39 | 2.44 ± 1.22 |
| CRP (mg/L) | 4.80 ± 0.97 | 2.40 ± 1.50 | 3.00 ± 1.00 | 1.10 ± 0.80 |
| Death reasons |  |  |  |  |
| Cancer | 28 (21.7) | 0 | 0 | 0 |
| Infectious disease | 19 (15.0) | 0 | 0 | 0 |
| CVD | 45 (34.9) | 0 | 0 | 0 |
| Neurological and psychiatric diseases | 13 (10.1) | 0 | 0 | 0 |
| MODS | 20 (15.5) | 0 | 0 | 0 |
| Trauma | 4 (2.9) | 0 | 0 | 0 |

**Abbreviations**: SD, stand deviation; BMI, body mass index; CVD, cardiovascular disease; HDL-C, high-density lipoprotein cholesterol; LDL-C, low-density lipoprotein cholesterol; CRP, C-reactive protein; MODS, multiple organ dysfunction syndrome.
