# Supplementary material for: The clinical value of PLR, NLR, and MLR in predicting mortality risk in hospitalized patients
Source: Front Pharmacol. 2025 Dec 3;16:1736947. doi: 10.3389/fphar.2025.1736947 (PMC12708535; doi:10.3389/fphar.2025.1736947)
Supplement: Supplementary file 2 [file Table2.docx]

**Supplementary Table 2**. Multivariable logistic regression analysis results after propensity score matching

| **Marker** | **β** | **SE** | **Wald** | **OR (95% CI)^a^** | **P-value** |
| --- | --- | --- | --- | --- | --- |
| **mortality group vs. kidney disease group** |  |  |  |  |  |
| PLR | 0.421 | 0.130 | 9.70 | 1.52 (1.16-1.99) | < 0.001 |
| NLR | 0.484 | 0.137 | 12.65 | 1.62 (1.23-2.13) | < 0.001 |
| MLR | 0.412 | 0.135 | 8.79 | 1.49 (1.15-1.93) | < 0.001 |
| **mortality group vs. rehabilitation group** |  |  |  |  |  |
| PLR | 0.522 | 0.146 | 12.86 | 1.68 (1.26-2.24) | < 0.001 |
| NLR | 0.621 | 0.153 | 15.99 | 1.86 (1.36-2.54) | < 0.001 |
| MLR | 0.724 | 0.162 | 19.05 | 2.05 (1.48-2.84) | < 0.001 |
| **mortality group vs. healthy group** |  |  |  |  |  |
| PLR | 0.658 | 0.168 | 15.98 | 1.93 (1.42-2.63) | < 0.001 |
| NLR | 0.683 | 0.167 | 16.59 | 1.97 (1.43-2.72) | < 0.001 |
| MLR | 0.841 | 0.171 | 23.04 | 2.32 (1.65-3.26) | < 0.001 |
| **^a^**Logistic regression analysis was adjusted for age, gender, BMI, hemoglobin, total cholesterol, triglyceride and comorbidities including history of cancer, history of CVD, history of diabetes, history of neurological disease.  **Abbreviations**: PLR, platelet-to-lymphocyte ratio; NLR, neutrophil-to-lymphocyte ratio; MLR, monocyte-to-lymphocyte ratio; OR, odd ratio; 95% CI, 95% confidence interval; BMI, body mass index; CVD, cardiovascular disease. | | | | | |
